# Spatial accessibility to pediatric primary care in Philadelphia: an area-level cross sectional analysis

**DOI:** 10.1186/s12939-019-0962-x

**Published:** 2019-05-24

**Authors:** Abigail E. Mudd, Yvonne L. Michael, Steven Melly, Kari Moore, Ana Diez-Roux, Christopher B. Forrest

**Affiliations:** 1 Dornsife School of Public Health, Drexel University, 3215 Market Street, Philadelphia, PA 19104 USA; 2 Applied Clinical Research Center, Children’s Hospital of Philadelphia, 3401 Civic Center Blvd, Philadelphia, PA 19104 USA

**Keywords:** Spatial accessibility, Pediatric primary care, Disparity

## Abstract

**Background:**

Pediatric primary care visits are a foundational element in the health maintenance of children. Differential access may be a driver of racial inequities in health. We hypothesized that pediatric primary care accessibility would be lowest in neighborhoods with higher proportion of non-Hispanic Black residents.

**Methods:**

Annual ratios (2008–2016) of providers to pediatric population were calculated by census tract in Philadelphia, Pennsylvania. Marginal logistic regression was used to estimate the independent association between neighborhood racial composition and access to pediatric primary care controlling for confounders.

**Results:**

In general, low access to care was associated with greater neighborhood disadvantage (e.g., SES, % poverty, % public insurance). After controlling for neighborhood indicators of disadvantage, risk of being in the lowest quintile of access significantly increased as the percent of non-Hispanic Black residents increased.

**Conclusion:**

A new measure of pediatric primary care accessibility demonstrates a persistent disparity in primary care access for predominantly non-Hispanic Black neighborhoods.

## Background

The foundation for the health of a population is forged during childhood. Key to ensuring healthy development is regular and accessible pediatric primary care. In primary care settings, children receive age-appropriate immunizations, screening for medical and developmental disorders, and treatment for acute and chronic conditions [1]. Ample evidence suggests that accessible primary care is necessary to maintain and improve the health of populations [2].

Access to care can be defined as an individual or population’s capacity to gain entry into the health system. It has spatial, organizational, and financial dimensions [3]. In an era of expanded financial accessibility through State Children’s Health Insurance Programs and the Affordable Care Act [4], increased emphasis should be placed on assuring other types of access, such as spatial accessibility to primary care. Prior research indicates that better spatial accessibility to primary care is associated with higher primary care service use and lower use of Emergency Departments, and a lower likelihood of preventable hospitalization [5, 6]. Prior qualitative research with parents living in low-income, urban areas identified significant environmental barriers to health care access for pediatric patients, including distance to primary care providers and lack of transportation, lack of money for transportation and medications, and lack of social support from friends and community. Additional barriers included providers that do not accept Medicaid, lack of availability of primary provider at times parents are available, and wait time to get an appointment [7].

Racial inequities in health may be driven by differential access to resources in the neighborhood environment, such as health care [8]. In the USA, compared to Whites, Non-Hispanic Blacks are disproportionately exposed to disadvantaged neighborhoods [9] and the dearth of positive features and excess of negative features associated with these environments [10]. Studies of spatial accessibility to pediatric care support a similar pattern among racially segregated neighborhoods [11, 12]. Prior research suggests an inverse association between spatial accessibility to adult care and a high proportion of Non-Hispanic Black residents at the neighborhood-level [13].

The majority of research has focused on adult accessibility or pediatric and adult primary care in the same analysis. Two prior studies [14, 15] evaluated pediatric-specific primary care accessibility in Washington D.C. using a previously generated spatial analysis dataset that used the American Medical Association’s Masterfile [11]. These data have measurement errors including variation in counts with slight changes in primary care definition, exclusion of important primary care providers, i.e., physician assistants and nurse practitioners, and outdated data [16]. Another recent study estimated access to pediatric asthma providers (allergists and pulmonologists only) by county for all of North Carolina and Georgia [17]. In more urban areas, county-wide access may be high but may obscure smaller area variation in access by census tract-level. Brown and colleagues described census tract-level variation in access to adult primary care within Philadelphia County [18]. These studies did not take road networks into account. Finally, none of these studies used historical data to estimate access over time.

We created a longitudinal measure of neighborhood-level accessibility to pediatric primary care for Philadelphia, Pennsylvania. As part of a larger study evaluating individual- and neighborhood-level characteristics associated with pediatric unplanned hospitalization, we evaluated spatial accessibility from 2008 to 2016. To better understand the possible role of health care access in racial inequities, we evaluated whether access to pediatric primary care differed by the proportion of Non-Hispanic Black residents in the neighborhood. Based on prior research [12], we hypothesized that pediatric primary care accessibility would be lowest in neighborhoods with a higher proportion of Non-Hispanic Black residents.

The institutional review boards of Children’s Hospital of Pennsylvania and Drexel University (Philadelphia, Pennsylvania) approved this research.

## Data, measurement, and methods

### Data

Office addresses of physicians (Doctorate of Medicine (MD) and Doctorate of Osteopathic Medicine (DO)), nurse practitioners (NP), and physician’s assistants (PA) with the specialties of internal medicine pediatrics, family medicine, pediatrician, or general practice in the metropolitan area of Philadelphia (includes Southeastern PA, Southern NJ, Delaware, and Northeastern MD) was obtained from SK&A Office-Based Providers Database, a product of SK&A Information Services, Irvine, CA, a healthcare database company.

Because SK&A data included historical data for some but not all public health centers [18], we collected additional data on the Federally Qualified Healthcare Centers (FQHCs) using a telephone survey. We called FQHCs a maximum of 3 times during business hours and requested information on the current number of MD, DO, NP, and PAs practicing pediatric primary care (defined as specialties listed as internal medicine pediatrics, family medicine, pediatrician, or general practice). The physical addresses of the FQHCs were collected from the Health Resources and Services Administration’s “Find a Health Center” tool. These data were supplemented by data on the FQHC’s website, if available.

SK&A data was used to define the provider counts at FQHCs when available. If SK&A data were available for a given FQHC for some years but not all, we used extrapolation from later years to impute the missing data. For example, if the SK&A dataset was missing provider counts for a FQHC in 2010 but included provider counts from 2009 and 2011, the 2011 data was used as an estimate for the count in 2010. If no SK&A data was available for a given FQHC, we used survey responses and extrapolated prior years based on current provider count. Survey data were used for a small minority of FQHCs that were missing completely in SK&A (*n* = 6, 9.8%).

### Measurement: access to pediatric primary care

Office addresses for practice locations were geocoded using ArcGIS (Geographic Information System) (version 10.5) and Esri world geolocator. Post Office (PO) boxes (*n* = 37, 0.76% of address data) were traced to physical practice location using the practice name, city, and Google maps. We used census tracts to represent neighborhoods. Philadelphia census tracts are relatively small with a median land coverage of 0.23 mile^2^ [19]. The 2010 Philadelphia County Census Tract centroids were mapped along the navigable street network. The Esri Business Analyst 2016 street network dataset, which includes data on typical driving times, was used to identify network buffers representing the area within a 5-min drive time from the location on the street network closest to each tract centroid [18]. If a practice location fell within this buffer, the residents of this neighborhood were assumed to have access to this location’s services. For each year, 2008–2016, we summed the total number of providers located within the buffer for each census tract. Following the Health and Services Administration’s estimates [20], we weighted NPs and PAs by 0.75 to account for smaller patient loads compared to MDs and DOs. To account for providers being shared between census tracts, the ratio of providers to child population was calculated as follows:

$$ =\frac{\left(\mathrm{total}\ \mathrm{providers}\ \mathrm{in}\ \mathrm{census}\ \mathrm{tract}\ \mathrm{buffer}\right)+\sum \left(\mathrm{total}\ \mathrm{providers}\ \mathrm{in}\ \mathrm{surrounding}\ \mathrm{census}\ \mathrm{tract}\ \mathrm{buffer}\mathrm{s}\right)}{\left(\mathrm{pediatric}\ \mathrm{pop}\ \mathrm{in}\ \mathrm{census}\ \mathrm{tract}\right)+\sum \left(\mathrm{pediatric}\ \mathrm{pop}\ \mathrm{in}\ \mathrm{surrounding}\ \mathrm{census}\ \mathrm{tract}\ \mathrm{buffer}\mathrm{s}\right)} $$

“Surrounding” was defined as any census tract that shared a border with the census tract of interest.

Finally, we excluded all census tracts in the lowest 5th percentile of pediatric population for any year 2008–2016 (*n* = 33). Because of the small number of children in these neighborhoods, observed changes could be due to a meaningful change in provider environment or a small change in pediatric population. Thus, our final sample of neighborhoods defined by census tract included 350 (91% of the total number of census tracts in Philadelphia).

### Measurement: racial segregation

We used census tract-level data available from the American Community Survey for neighborhood-level characteristics including racial/ethnic distribution. Specifically, we assessed percent of residents by race/ethnicity: non-Hispanic Black, Hispanic, non-Hispanic Asian.

### Measurement: neighborhood covariates

We selected neighborhood-level covariates based on prior research linking these factors to health care access in order to adjust for confounding in our models (described below) [6, 21, 22]. Neighborhood-level characteristics were assessed using American Community Survey: age distribution, education attainment, single parent households, crowding, Gini index of income inequality, median household income, public assistance, poverty, unemployment, and vehicle availability. We used a socioeconomic status (SES) score developed in prior research [23] and commonly used in neighborhood research [24]. SES score was compiled from 6 unweighted variables from the American Community Survey: (1) median value of occupied housing units, (2) % persons 25 years of age and older with a high school education or more, (3) % persons 25 years of age and older with a Bachelor’s degree or more, (4) % residents with management, professional, or related occupation, (5) median household income, (6) % households with interest, dividends, or net rental income. Median value of occupied home and median household income were log transformed then the six variables were summed. The sum of the 6 variables was transformed into a z-score with higher values indicating higher SES [23]. Data on percentage of pediatric (under 18 years of age) residents with health care insurance (private, public, or none) was obtained from the American Community Survey. Data on rate of violent crime and drug offenses per 10,000 residents was obtained from police reported data, available on OpenDataPhilly [25].

We standardized neighborhood characteristics using z-scores with mean 0 and standard deviation of 1.

### Statistical analysis

Descriptive statistics (means and proportions) were used to examine spatial accessibility to pediatric primary care overall and by quintiles across years (2008–2016). A Kruskal-Wallis test was used to assess stability of ratios between 2008 and 2016 by quintile of spatial accessibility. In order to evaluate whether the neighborhood-level covariates were associated with access to pediatric primary care, we summarized neighborhood-level characteristics by quintile of spatial accessibility. An unpaired t-test was used determine significant differences in neighborhood-level covariates between neighborhoods with the highest versus lowest spatial accessibility.

We pooled data across years and modeled the odds of being in the lowest quintile of access (vs being in any of the other quintiles) as a function of each neighborhood characteristic. Given the nested nature of the data, we estimated the independent association between neighborhood racial composition and access to pediatric primary care controlling for covariates using Generalized Estimating Equations (GEE) marginal logistic models that accounted for within-tract correlations over time. Our null hypothesis was that there was no association between spatial accessibility and proportion of Non-Hispanic Black residents.

To create the most parsimonious multivariable model estimating the association between racial segregation and access to care adjusted for confounding neighborhood factors, we used a backward step-wise variable selection approach. All neighborhood characteristics (see Table 1) were included in an initial model. Each sequential model dropped the variable with the highest *p*-value until only variables with *p* < 0.1 were retained. To evaluate additional potential confounding factors, dropped variables were introduced in one at a time and retained if their addition resulted in a 10% or greater change in the effect estimate for percent of Non-Hispanic Black residents.

**Table 1:** Mean, mean difference, and percent difference of neighborhood-level characteristics of the first quintile (Q1, lowest access) and fifth quintile (Q5, highest access) of spatial accessibility to pediatric primary care in 2015 Philadelphia, PA

Neighborhood variable	Spatial access Q1	Spatial access Q5	Q5-Q1	% Difference^a^
% non-Hispanic Black	0.55	0.24	−0.31	129.2*
% Hispanic	0.07	0.06	−0.01	16.7
% non-Hispanic Asian	0.03	0.06	0.30	50.0
SES Factor Score^b^	−4.96	0.825	5.80	701.2*
% < 5 years of age	0.08	0.06	−0.02	33.3
% < 18 years of age	0.26	0.18	− 0.08	44.4
% persons with high school education or more	0.80	0.86	0.06	7.0
median household income	34,564.5	42,750.0	8185	19.1
% households with public assistance	0.10	0.06	−0.03	66.7
Gini Index of income inequality^c^	0.44	0.49	0.05	10.2
% persons below poverty level (all ages)	0.27	0.27	0.00	0.0
% persons less than 18 below poverty level	0.40	0.21	−0.19	90.5*
% under 18 with private health insurance only	0.30	0.49	0.19	38.8
% under 18 with public health insurance only	0.61	0.39	−0.22	56.4*
% under 18 with no health insurance coverage	0.03	0.03	0.00	0.0
% unemployed	0.17	0.11	−0.06	54.5*
% single parent household with children under 18	0.21	0.10	−0.11	110.0*
% of occupied housing units with > 1 person per room	0.02	0.02	−0.00	0.0
Density of violent crime per 10,000 people	259.9	184.7	−75.2	40.7
Density of drug offenses per 10,000 people	26.3	12.7	−13.6	107.1*
% housing units with no vehicle available	0.33	0.36	0.03	8.3

*significant difference of means at *p* = 0.05 using unpaired t-test

^a^% difference = |Q5 – Q1|/ Q5

^b^non-weighted score based on 6 measures: (1) median value of occupied housing units, (2) % persons 25 years of age and older with a high school education or more, (3) % persons 25 years of age and older with a Bachelor’s degree or more, (4) % residents with management, professional, or related occupation, (5) median household income, (6) % house with interest, dividends, or net rental income

^c^summarizes the allocation of money in an area. 0 corresponds to perfectly equal income distribution among residents. 1 corresponds to perfect inequality, a single resident receiving all the income for the area [26]

## Results

The ratio of providers to children was skewed to the right meaning that some neighborhoods have extremely high accessibility compared to the majority of the city. Over the period 2008–2016, the median accessibility by neighborhood was 3.7 per 1000 children (IQR 2.1, 6.9).

**Fig. 1:**
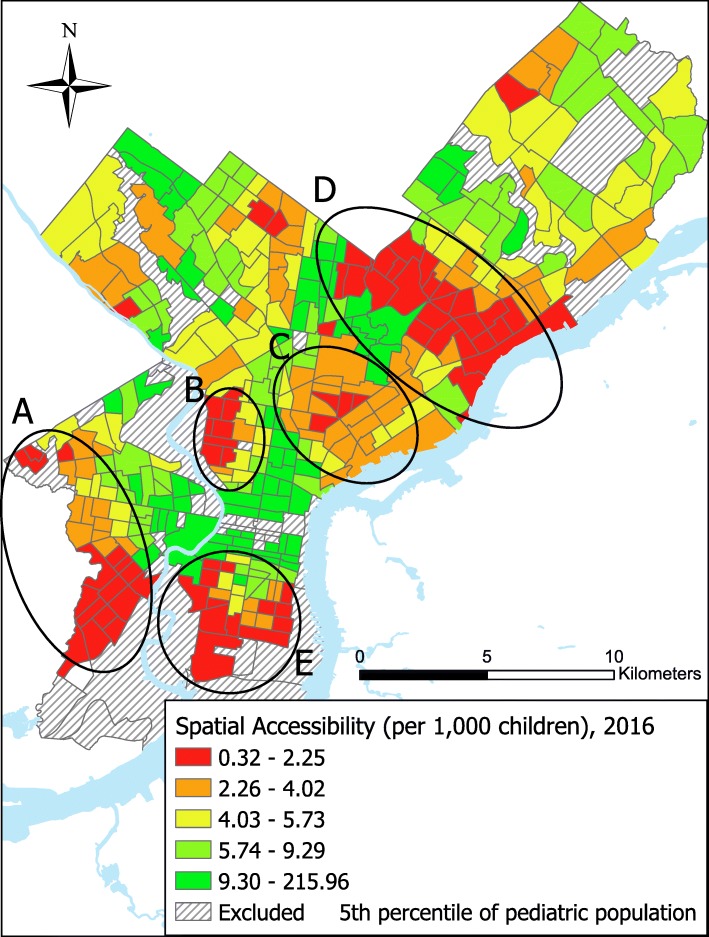
Census tract spatial accessibility to pediatric primary care in quintiles in Philadelphia, PA 2016. The areas of lowest access based on qualitative analysis are identified by circles **a**-**e**. These are similar to the areas identified as clusters of lowest access to adult primary care in Philadelphia by Brown and colleagues [18]

Accessibility to pediatric primary care in Philadelphia for 2016 is summarized in Fig. 1. We identified 5 clusters of lowest access, defined as 5 continuous census tracts in the 2 lowest quintiles of access with at least 4 census tracts being in the lowest quintile, distributed throughout the city. These areas are similar to clusters of low adult primary care access identified by Brown and colleagues [18]. The distribution of spatial accessibility was consistent overtime, with no differences by year by quintiles (*p* > 0.05).

Neighborhoods with the lowest access to pediatric primary care had the greatest proportion of Non-Hispanic Black residents; the percent decreased by 132% comparing the proportion in the neighborhoods with the lowest access to the neighborhoods with the highest access (Table 1). Many neighborhood-level indicators of socioeconomic position also varied significantly by spatial accessibility to pediatric primary care, including SES index, percent below poverty, and percent of children with public health insurance. After adjustment for SES index, income inequality, proportion of residents under 5, health insurance status, vehicle access, and crime, a one standard deviation increase in percent Non-Hispanic Black within a neighborhood was associated with a 52 % (52%) greater odds of being in the lowest quintile of spatial accessibility (95% confidence interval 1.04–2.22) (Table 2).

**Table 2:** Adjusted odds ratios of being in the lowest quintile of spatial accessibility to pediatric primary care associated with neighborhood variables in Philadelphia, PA 2008–2016

Neighborhood variable (per SD increase)	AOR	95% CI
% non-Hispanic Black*	1.52	1.04–2.22
% Hispanic^a^	0.96	0.70–1.31
% non-Hispanic Asian*	1.28	1.01–1.64
SES Factor Score – non-weighted score based on 6 measures*	0.39	0.21–0.75
% < 5 years of age^a^	1.15	0.98–1.34
Gini Index of inequality*	0.55	0.42–0.71
% under 18 with public health insurance only^a^	1.13	0.83–1.53
% under 18 with no health insurance coverage^a^	1.04	0.87–1.24
Density per 10,000 population for violent crime^a^	1.08	0.83–1.40
% housing units with no vehicle available*	0.65	0.47–0.91

*significant at *p* = 0.05

^a^reintroduced confounder

## Discussion

While Philadelphia has some areas of dense provider coverage (particularly around large pediatric medical institutions), most of the city’s children live in neighborhoods of relatively low accessibility. Residents of these neighborhoods have to travel farther to access pediatric primary care. Areas of lowest access to pediatric primary care were similar to the areas of limited spatial accessibility to adult primary care in Philadelphia observed by Brown and colleagues [18].

We observed a consistent racial disparity in pediatric access for the Non-Hispanic Black community of Philadelphia consistent with prior findings of disparities in adult primary care accessibility [13]. Our findings are also consistent with prior research that report majority-Hispanic neighborhoods fare better than their Non-Hispanic Black counterparts [12]. The more equitable access for Hispanic population in this study may reflect a network of FQHCs specifically placed in dense Hispanic neighborhoods.

The measure has several important limitations to note. Imputation was necessary for NPs and PAs for 2008–2009 which could have affected the estimated provider to population ratios particularly in low access census tracts. The measure of spatial accessibility implicitly assumes that caregivers seek care for children close to their home as opposed to where they work or where their children go to school. This measure does not take public transportation into account. The 5-min drive-time radius technically assumes that individuals will be driving to their appointments. However, the radius is useful to define areas that would be reasonably easy to access by alternative transport. Additionally, this analysis is cross-sectional and thus we can make no conclusion on the cause of the observed correlation between low spatial accessibility and Non-Hispanic Black neighborhoods. Additionally, we did not collect information from residents of these neighborhoods that would allow us to better understand the ways in which spatial access influences care seeking. While we adjusted for SES in our models using a commonly used index that includes a number of neighborhood-based indicators of socio-economic status [23], we cannot rule out the possibility of residual confounding of the estimates as a result of some error in our measurement of SES.

## Conclusions

We identified areas of limited access in Philadelphia which were more common in neighborhoods with larger proportions of Non-Hispanic Black residents. Areas with limited care sites relative to the pediatric population may benefit from targeted placement of primary care, such as FQHCs. Future research is needed to investigate if this measure is associated with disparities in individual-level health care utilization and to evaluate mechanisms through which spatial access works to influence disparities in utilization and health outcomes.

## Abbreviations

DO: Doctorate of Osteopathic Medicine
FQHC: Federally Qualified Healthcare Center
GEE: Generalized Estimating Equations
GIS: Geographic information systems
MD: Doctor of Medicine
NP: Nurse practitioner
PA: Physician’s assistant
SES: Socio-economic status
